# Adapting the acceptance of illness scale for Sinhala-speaking populations: a psychometric study

**DOI:** 10.3389/fpsyg.2026.1889860

**Published:** 2026-09-23

**Authors:** Darshika Thejani Bulathwatta, Judyta Borchet, Asanka Bulathwatta, Monika Bidzan-Wiącek, Sebastian Mueller-Haugk, Ilona Bidzan-Bluma, Marcus Stueck, Mariola Bidzan

**Affiliations:** 1 Department of Psychology and Counseling, Faculty of Health Sciences, The Open University of Sri Lanka, Colombo, Sri Lanka; 2 Institute of Psychology, Faculty of Social Sciences, University of Gdańsk, Gdańsk, Poland; 3 Academic Center for Psychological Support, University of Gdańsk, Gdańsk, Poland; 4 Institute of Pedagogy and Languages, University of Applied Sciences in Elbląg, Elbląg, Poland; 5 Department of Psychology, Faculty of Arts, University of Peradeniya, Peradeniya, Sri Lanka; 6 Institute of Psychology, University College of Professional Education, Wrocław, Poland; 7 Division of Quality of Life Research, Faculty of Health Sciences with the Institute of Maritime and Tropical Medicine, Medical University of Gdańsk, Gdańsk, Poland; 8 Duale Hochschule Sachsen, Standort Plauen, Plauen, Germany; 9 International Biocentric Research Academy, Leipzig, Germany

**Keywords:** acceptance of illness scale, chronic illness, cross-cultural adaptation, psychological assessment, Sinhala language

## Abstract

**Background:**

Acceptance of illness is an important psychological process that influences how individuals adapt to the challenges and limitations associated with chronic diseases. Greater illness acceptance has been associated with better psychological adjustment, improved coping, and enhanced wellbeing among individuals living with chronic conditions. The Acceptance of Illness Scale (AIS) is widely used to assess individuals’ psychological adaptation to chronic disease. However, a validated Sinhala version has not been available, limiting its use in Sri Lanka. This study aimed to culturally adapt and evaluate the psychometric properties of the Sinhala version of the AIS among individuals living with chronic illnesses.

**Methods:**

The original AIS was translated into Sinhalese using the back-translation process. Data were collected from 141 adults (63.1% women) who self-identified as people with chronic diseases, spoke Sinhala, and were living in Sri Lanka. Principal component analysis (PCA) and confirmatory factor analysis (CFA) were conducted to evaluate the scale’s dimensional structure. Cronbach’s α was used to assess internal consistency, and r Pearson correlations with measures of life satisfaction and depression were employed to examine convergent validity.

**Results:**

The Sinhala AIS demonstrated satisfactory internal consistency (Cronbach’s α = 0.75). PCA supported a single-component structure consistent with the original version, explaining 52.66% of the total variance. Subsequent CFA conducted on the same sample provided partial support for the one-factor structure (CFI = 0.99, AGFI = 0.97, TLI = 0.99, SRMR = 0.06, RMSEA = 0.097). Acceptance of illness was positively correlated with life satisfaction (*r* = 0.253) and negatively correlated with depression (*r* = −0.336), providing preliminary evidence for construct validity.

**Conclusion:**

The findings provide preliminary support for the reliability and construct validity of the Sinhala AIS among Sinhala-speaking adults with chronic diseases and encourage further research on its psychometric properties and applicability in diverse clinical populations. The scale may be a useful tool for research and potentially for clinical applications; however, further validation is recommended.

## Introduction

Chronic diseases pose a major challenge to population health across the lifespan. Despite global public health efforts, chronic and non-communicable diseases (NCDs) such as cancer, cardiovascular and metabolic conditions, and mental health disorders continue to increase worldwide (Thomas et al., 2023). Chronic diseases are defined as long-term conditions lasting at least one year that require ongoing medical care or limit an individual’s ability to perform daily activities (Centers for Disease Control Prevention, 2024). The onset and progression of chronic diseases are influenced by multiple interacting determinants, including social and economic conditions, health behaviors, access to healthcare, and environmental factors. These social determinants of health contribute substantially to disparities in chronic disease risk and outcomes (Hacker et al., 2021). In 2021, NCDs accounted for approximately 43 million deaths, representing 75% of all non-pandemic-related deaths globally. Cardiovascular diseases were the leading cause (about 19 million deaths), followed by cancers (10 million), chronic respiratory diseases (4 million), and diabetes, including diabetes-related kidney disease (over 2 million deaths) (World Health Organization, 2024). In addition to biomedical determinants, psychosocial factors-including emotional responses, social support, health-related beliefs-play an important role in how individuals cope with and manage chronic conditions (Jiakponna et al., 2024).

### Chronic diseases in Sri Lanka

Non-communicable diseases (NCDs) are the leading cause of mortality in Sri Lanka, accounting for approximately 83% of all deaths nationwide (Epidemiology Unit Ministry of Health, 2025). A substantial proportion of premature mortality is attributable to modifiable behavioral risk factors, including tobacco use, unhealthy diets, harmful alcohol consumption, and physical inactivity (Ministry of Health Sri Lanka, 2022). Cardiovascular diseases, diabetes, chronic respiratory diseases such as asthma and chronic obstructive pulmonary disease (COPD), and chronic kidney disease (CKD) are among the most prevalent chronic conditions in Sri Lanka (Ministry of Health Sri Lanka, 2022). In 2021, 18 million people died from NCDs before the age of 70, with 82% of these premature deaths and 73% of all NCD-related deaths occurring in low- and middle-income countries such as Sri Lanka (World Health Organization, 2024). Since independence, Sri Lanka has achieved notable gains in communicable disease control, maternal and child health, and the near eradication of vaccine-preventable diseases. However, NCDs have now overtaken communicable diseases as the leading contributors to mortality, morbidity, and disability in the country (Ministry of Health Sri Lanka, 2022). Nevertheless, communicable diseases continue to pose important public health challenges in Sri Lanka. Dengue remains one of the country’s most significant vector-borne diseases, with recurrent outbreaks placing considerable demands on the healthcare system. Furthermore, the COVID-19 pandemic highlighted the ongoing vulnerability of the health system to emerging infectious diseases and substantially influenced patterns of infectious disease transmission and healthcare delivery. Although the burden of non-communicable diseases now exceeds that of communicable diseases, these concurrent health challenges illustrate Sri Lanka’s continuing double burden of disease and the need for a healthcare system capable of addressing both acute infectious diseases and long-term chronic conditions (Ariyaratne et al., 2022).

A study conducted in Sri Lanka reported that individuals with chronic illnesses generally have lower income and incur higher out-of-pocket health expenditures than those without such conditions (Jayathilaka et al., 2020). The study identified gender, age, employment status, education level, and urban residence as key factors associated with a higher risk of chronic illness, which can reduce economic stability. Chronic diseases contribute substantially to income insecurity, particularly among socioeconomically vulnerable groups, underscoring the need for accessible healthcare financing and supportive policy responses (Kumara and Samaratunge, 2017). Furthermore, a qualitative study in rural Sri Lanka found that limited infrastructure, together with social and cultural influences, constrained physical activity among individuals with type 2 diabetes (Medagama and Galgomuwa, 2018). In this study, participants often struggled to distinguish between routine household chores and intentional exercise while also showing limited awareness of appropriate physical activity. Reported barriers included health problems, time constraints, environmental challenges, prioritization of domestic responsibilities, cultural norms (e.g., social embarrassment), and stigma, all contributing to reduced physical activity and poorer diabetes management in rural communities.

Sri Lanka provides free universal healthcare through a well-established system of preventive and curative services. However, demographic and epidemiological transitions have increased the burden of NCDs, placing considerable strain on healthcare infrastructure (Wanigasuriya, 2019). Key challenges include limited access to acute care for conditions such as strokes and heart attacks, insufficient diagnostic facilities, and overcrowded tertiary hospitals. These pressures are further increased by the growing demand for long-term care and disease management associated with the increasing burden of NCDs across adulthood. The Sri Lankan healthcare system therefore faces significant challenges in addressing the psychosocial and healthcare needs of individuals living with chronic conditions.

### Acceptance of illness

Acceptance is considered a key indicator of how well individuals adapt to living with a chronic illness and contributes to maintaining independence (Obiegło et al., 2016). In this context, acceptance reflects a patient’s ability to acknowledge the reality of their condition, including its limitations and losses. It represents a crucial psychological shift that fosters emotional stability and supports adaptation. Achieving this state is central to the therapeutic process, as it enhances mental resilience and positively affects how individuals cope with daily life and treatment (Wysokiński et al., 2018). Furthermore, lower levels of illness acceptance have been associated with poorer psychological adjustment, particularly higher symptoms of depression and anxiety among individuals with chronic disease (Uchmanowicz et al., 2016). Higher acceptance of illness has also been linked to greater life satisfaction, suggesting that individuals who more fully accept their condition report higher satisfaction with life (Kocatepe et al., 2020; Kowalewska et al., 2020). Acceptance of a chronic illness is influenced by social support, particularly from close family and healthcare providers. Such support helps maintain patient dignity and promotes understanding of the disease, its course and prognosis, as well as the patient’s mental and general health status and socio-demographic factors (Oskedra et al., 2021). Therefore, Clinicians should consider the extent to which patients have accepted their illness and address any barriers to acceptance (Oskedra et al., 2021). Additionally, the gender and education influence the level of disease acceptance, though they do not appear to affect quality of life (Szpilewska et al., 2018).

The Acceptance of Illness Scale (AIS), developed by Felton et al. (1984), is one of the most widely used instruments for assessing psychological adaptation to chronic illness. This scale evaluates the extent to which individuals accept the physical, emotional, and social consequences of living with a chronic condition. Owing to its brevity, ease of administration, and robust psychometric properties, the AIS has been translated and validated in numerous languages and has been applied across diverse chronic disease populations (Czerw et al., 2022; Leong et al., 2021; Centers for Disease Control Prevention, 2024). However, evidence from one cultural context cannot be assumed to apply directly to another, highlighting the need for cross-cultural adaptation and psychometric validation before use in a new linguistic and cultural setting.

### Rationale for the study

The AIS was selected for this study because it is one of the most widely used and extensively validated instruments for assessing illness acceptance across a broad range of chronic diseases in multiple countries. Its brevity, ease of administration, and robust psychometric performance make it suitable for both clinical and research settings.

Cross-cultural adaptation involves more than linguistic translation. Cultural differences in illness beliefs, emotional expression, family relationships, and perceptions of dependence may influence how individuals interpret questionnaire items. Therefore, before the AIS can be used in a new cultural context, its adaptation was necessary.

In Sri Lanka, chronic diseases continue to increase, yet no validated Sinhala version of the AIS is available for assessing illness acceptance among Sinhala-speaking adults. Acceptance of illness has been shown to influence psychological adaptation, emotional stability, coping strategies, and treatment outcomes (Obiegło et al., 2016; Oskedra et al., 2021; Wysokiński et al., 2018). However, the absence of a culturally adapted and psychometrically validated Sinhala version limits clinicians’ ability to assess acceptance using a standardized tool and restricts researchers’ ability to evaluate psychosocial adaptation and compare findings across studies. A multi-country study of advanced cancer patients in Asia, including Sri Lanka, highlighted the importance of assessing psychological outcomes alongside prognostic awareness and demonstrated that acceptance of illness can moderate the impact of prognosis on anxiety, depression, and spiritual wellbeing (Ozdemir et al., 2022). Nevertheless, in Sri Lanka, the study lacked standardized measures for assessing acceptance, illustrating a critical gap in available assessment tools.

### Study objective

The aim of this study was to translate and culturally adapt a Sinhala version of the Acceptance of Illness Scale (AIS) for Sinhala-speaking adults with chronic diseases living in Sri Lanka. Developing a Sinhala version of the AIS would provide a culturally appropriate instrument to measure acceptance of illness in the target population accurately, thereby enabling better psychosocial support, targeted interventions, and improved health outcomes for patients with chronic diseases, as well as inclusion of Sinhala-speaking samples in international studies employing the AIS.

## Materials and methods

Prior to the start of the study, ethical approval was obtained from the Ethics Review Board of the (blinded for review). All participants were volunteers, and informed consent was obtained from each participant before they were granted access to the online survey. Eligibility was based on self-identification as a person living with at least one chronic disease. In the recruitment materials, participation was restricted to individuals who considered themselves to have a chronic illness; however, no information on diagnosis, disease type, duration, or medical verification was collected.

### Procedure

The study enrolled individuals residing in Sri Lanka who were fluent in the Sinhala language and self-identified as having at least one chronic disease. Data were collected through an online survey administered via Google Forms from September 2023 to June 2025.

The survey was distributed among: (1) undergraduate and postgraduate students affiliated with four Sri Lankan universities, which represent students from different areas of the country, (2) school leavers who completed the General Certificate of Education Advanced Level (GCE A/L), and (3) personal contacts (i.e., snowball sampling), which led to gathering responses from individuals outside the university context, including some who were employed in the public sector (these participants were not recruited as a predefined target group). The distribution of the invitation to join the study was carried out through various channels, including university email lists, official social media pages, and professional networks, to ensure broad accessibility across different regions of Sri Lanka and to minimize the selection bias.

Participation in the study was voluntary, and informed consent was obtained electronically before participants could access the survey. The consent form, provided in Sinhala, detailed the study’s purpose, procedures, potential risks and benefits, and confidentiality assurances emphasizing that participants’ responses would remain anonymous and used solely for research purposes. The survey was designed to be completed in approximately 15 min, and participants were encouraged to answer all questions honestly and thoughtfully.

### Participants

A total of 278 Sinhala-speaking adults living in Sri Lanka initially joined the study. Of these, 141 participants met the inclusion criteria (i.e., self-identifying as having at least one chronic illness) and were included in the final data analysis.

The final study sample consisted mainly of women (*n* = 89; 63.1%), while men accounted for 36.9% (*n* = 52). Participants’ age ranged from 18 to 65 years. The most frequent age group was 18–25 years, whereas the least frequent age group was 56–65 years. The age group distribution is presented in Table 1.

**TABLE 1:** Participants’ age groups (*N* = 141).

Age group	Frequency	Percentage
18–25	45	31.9
26–35	27	19.1
36–45	30	21.3
46–55	32	22.7
56–65	7	5.0

Dominant religion among study participants was Buddhism (*n* = 134, 95%), followed by Catholicism (*n* = 3, 2.1%), Hindu (*n* = 2, 1.4%), and Atheism (*n* = 2, 1.4%). With regard to marital status, the sample was balanced, as 51.1% were married (*n* = 72) and 48.9% were unmarried (*n* = 69). Participants reported different levels of educational attainment. The largest group consisted of undergraduate students (*n* = 49; 34.8%), followed by graduates (*n* = 41; 29.1%) and postgraduates (*n* = 38; 27.0%). A smaller proportion of participants reported completing the General Certificate of Education (G.C.E.) Advanced Level (A/L), combining two subcategories for clarity (*n* = 11; 7.8%) or the General Certificate of Education (G.C.E) Ordinary Level (O/L) (*n* = 2; 1.4%). In the Sri Lankan education system, O/L and A/L represent national secondary school examinations typically completed before entering higher education. The distribution of participants across education levels is presented in Table 2.

**TABLE 2:** Participants’ education (*N* = 141).

Education	Frequency	Percentage
A/L	9	6.4
GCE advanced level	2	1.4
Graduate	41	29.1
O/L	2	1.4
Post graduate	38	27.0
Under graduate	49	34.8

### Measures

The study consisted of a researcher-designed demographic questionnaire, culturally adapted Sinhala version of the Satisfaction with Life Scale (Diener et al., 1985; Rajapaksha et al., 2025), the Sinhala adaptation of the Beck Depression Inventory (Beck et al., 1988; Rodrigo et al., 2015), and the authors’ Sinhala translation of the Acceptance of Illness Scale.

#### Socio-demographic information

The survey included a demographic information sheet that collected data on participants’ age, gender, religion, marital status, and educational background. All these variables were categorical.

#### The Satisfaction with Life Scale (SWLS)

The SWLS is a widely used measure of global life satisfaction, developed to assess the cognitive component of subjective wellbeing (Diener et al., 1985). It is a five-item scale, with responses rated on a seven-point Likert-type scale ranging from strongly disagree to strongly agree. The scale is unidimensional and has demonstrated strong psychometric properties across diverse populations (Pavot and Diener, 2008). The SWLS has previously been used among Sinhala-speaking populations in Sri Lanka, including caregivers (Rodrigo et al., 2013), medical students (De Zoysa et al., 2015), and healthcare workers (Wijayatunga, 2021). In the present study, the SWLS was administered in Sinhala (Rajapaksha et al., 2025). Reliability analysis indicated adequate internal consistency in the current sample (α = 0.84).

#### The Beck Depression Inventory (BDI)

The BDI is a widely used self-report tool designed to measure the severity of depressive symptoms (Bautovich et al., 2018). It consists of 21 items rated on a four-point Likert-type scale (0–3), with total scores ranging from 0 to 63. The scale has demonstrated good internal consistency (α ≈ 0.87) (González-Flores et al., 2021; Hacker et al., 2021) and has been frequently applied in both clinical and non-clinical populations, including patients with chronic kidney disease (Richter et al., 1998). This study employed the Sinhala adaptation of the BDI-II (Rodrigo et al., 2015). Reliability analysis indicated adequate internal consistency in the current sample (α = 0.90).

#### Acceptance of Illness Scale (AIS)

Acceptance of Illness Scale is a widely used measure for assessing the extent to which individuals adapt to and accept the limitations associated with chronic illness (Felton et al., 1984). The tool consists of eight statements, each rated on a five-point Likert scale that captures varying levels of acceptance (Felton et al., 1984). The response options are anchored as follows: 1 = very poor acceptance, 2 = poor acceptance, 3 = average acceptance, 4 = acceptance, and 5 = full acceptance of illness.

A distinctive feature of the AIS lies in its directionality of items. The majority of statements are framed negatively, describing adverse consequences of illness (e.g., dependency, reduced self-esteem), and are scored in a straightforward manner, with higher values (closer to 5) reflecting a greater level of illness acceptance (Felton et al., 1984). However, item 6 is phrased in the opposite (positive) direction, and therefore requires reverse scoring. In this case, a response of “strongly agree” is scored as 1, while “strongly disagree” is scored as 5, ensuring consistency across the overall scoring structure.

The total score is derived by summing the eight items, yielding a possible range from 8 to 40 (Felton et al., 1984). Lower scores (close to 8) indicate extremely poor acceptance and adjustment, while higher scores (approaching 40) signify extremely high acceptance and adjustment. This scoring method allows the AIS to capture individual differences in psychosocial adaptation to illness sensitively.

### Translation procedure

Before initiating the adaptation process, the principal investigator obtained explicit permission from Tracey Revenson, one of the authors of the AIS, to use and adapt the instrument (T. A. Revenson, personal communication, 28 August 2023). The adaptation process was based on established cross-cultural translation procedures emphasizing independent forward translation, reconciliation, and back-translation, as described by Banville et al. (2000). No formal expert-panel review, cognitive interviewing, or pre-testing with representatives of the target population was conducted prior to data collection.

The translation process involved back-translation, whereby the instrument was first translated into Sinhala and subsequently translated back into English, following recommended procedures (Sireci and Berberoglu, 2000). There were four people involved in the translation process. Three Sinhala-speaking scholars with psychology degrees and English fluency independently translated the AIS into Sinhala. The translators then reconciled their translations and agreed on a preliminary Sinhala version. This version was subsequently back-translated into English by an independent bilingual scholar proficient in both Sinhala and English and who had not seen the original instrument. The back-translated version was compared with the original English version, and final minor revisions were made to the Sinhala version. No items required substantial modification, deletion, or replacement during the translation process, as the translators reached consensus regarding the semantic equivalence of all AIS items (see Supplementary material).

### Planned data analysis

Missing data were handled by excluding incomplete responses from analyses. Statistical analyses such as principal component analysis (PCA), reliability assessment, and correlation analysis were conducted using SPSS version 29. For the confirmatory factor analysis, JASP 0.97 was used. Because both PCA and CFA were conducted on the same sample, CFA was treated as a supplementary assessment of model fit rather than as an independent cross-validation of the identified structure.

### Construct validity

Data were analyzed using both principal component analysis (PCA) and confirmatory factor analysis (CFA) to evaluate the psychometric properties of the Sinhala version of the AIS. Prior to conducting the PCA, the suitability of the data for dimension reduction was assessed using the Kaiser-Meyer-Olkin (KMO) test of sampling adequacy and Bartlett’s test of sphericity. Components were retained based on eigenvalues, inspection of the scree plot, and theoretical interpretability of the solution.

Following PCA, a confirmatory factor analysis (CFA) was performed. The model fit was evaluated using the RMSEA, SRMR, CFI, TLI, and AGFI model fit indices. The study did not employ a cross-validation method. Because both PCA and CFA were conducted on the same sample of participants (*N* = 141), the CFA served as a complementary assessment of model fit. rather than as an independent cross-validation of the factor structure.

### Reliability and convergent validity

The internal consistency reliability of the scale was examined using Cronbach’s alpha coefficient. Convergent validity was evaluated by examining Pearson’s correlations (r) between AIS scores and theoretically related constructs. Associations with life satisfaction and depressive symptoms were expected based on previous literature.

## Results

### Exploratory factor analysis of the Sinhala version of the AIS

A PCA was conducted to explore the underlying structure of the Sinhala version of the AIS. The KMO measure of sampling adequacy was 0.88, indicating that the sample was suitable for factor analysis. Bartlett’s test of sphericity was significant (*p* < 0.001), confirming that the correlation matrix was appropriate for factor extraction. Examination of eigenvalues and inspection of the scree plot supported the retention of a single factor. This factor explained 52.66% of the total variance. The one-factor solution was theoretically consistent with the original version of the AIS and was interpreted as representing illness acceptance.

This finding suggests that all items measure a single underlying construct representing illness acceptance (see Figure 1).

**FIGURE 1:**
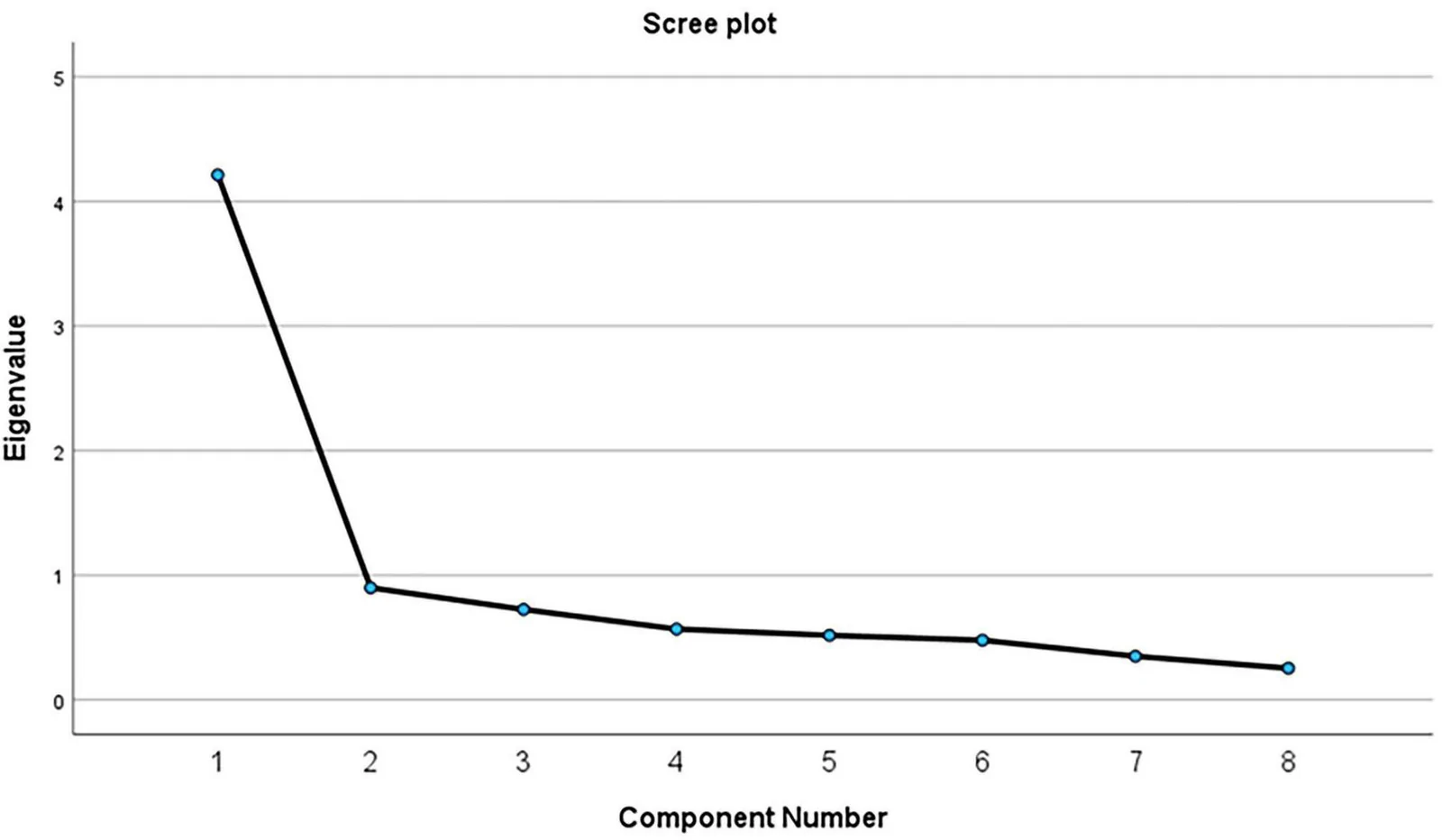
Scree plot for the Sinhala version of the Acceptance of Illness Scale (AIS).

### Confirmatory factor analysis of the Sinhala version of the AIS

We conducted a confirmatory factor analysis (CFA) of the Sinhala version of the AIS using the weighted least squares mean and variance-adjusted (WLSMV) estimator, appropriate for ordinal indicators (Li, 2016). The CFA tested the one-factor solution identified in the PCA. The model demonstrated a mixed pattern of fit indices. The chi-square test was statistically significant, χ^2^(20) = 46.10, *p* < 0.001, suggesting some degree of model misfit. However, incremental fit indices indicated good fit (CFI = 0.99, TLI = 0.99, AGFI = 0.97; values > 0.90 indicate adequate fit) (Hu and Bentler, 1999), and the standardized root mean square residual was acceptable (SRMR = 0.06; values ≤ 0.08 indicate adequate fit). In contrast, the root mean square error of approximation suggested only marginal fit (RMSEA = 0.097, 95% CI [0.061, 0.134]). Despite RMSEA not being fully satisfactory, the literature suggests it might be inflated due to small sample size (Kenny et al., 2015). Furthermore, factor loadings ranged from 0.52 to 0.84, confirming that all eight items contributed meaningfully to the measurement of the overall construct of acceptance of illness (see Figure 2). Taken together, these findings provide partial support for the one-factor structure of the Sinhala AIS rather than unequivocal confirmation of the model.

**FIGURE 2:**
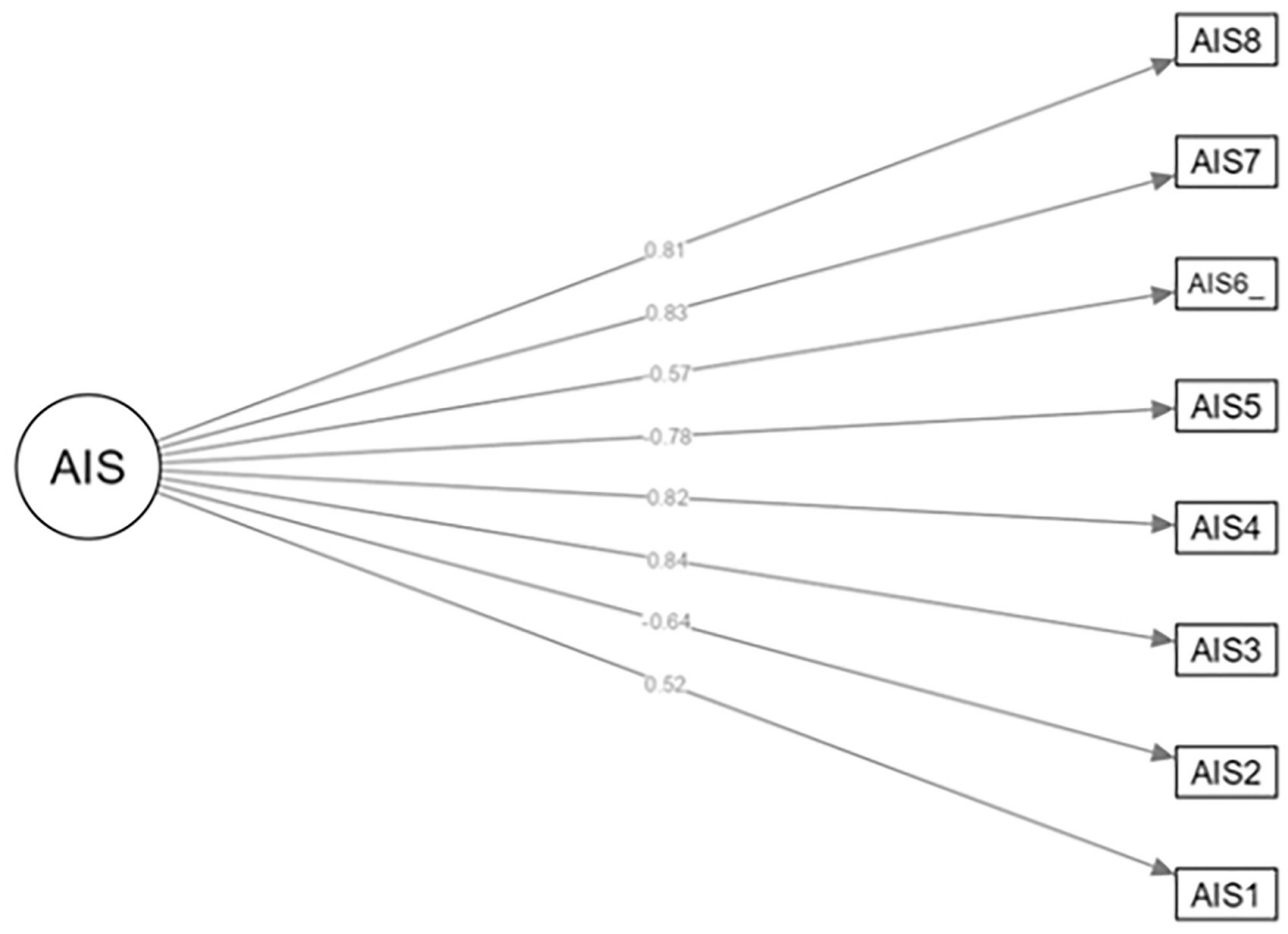
Factor structure of the Sinhala (AIS).

### Reliability and external validity

To assess the reliability of the adapted Sinhala version of the AIS, we adopted an *a priori* criterion that a Cronbach’s alpha (α) coefficient equal to or greater than 0.70 was satisfactory (Worthington and Whittaker, 2006). The adapted Sinhala version of the AIS demonstrated satisfactory reliability with α = 0.75, indicating good internal consistency.

To test the validity of the scores produced from the Sinhala version of the AIS, the following measures were used: the Sinhala adaptation of the Satisfaction with Life Scale (Rajapaksha et al., 2025) and the Sinhala adaptation of the Beck Depression Inventory (Rodrigo et al., 2015). Pearson’s correlation (*r*) analysis presented that the AIS score was positively associated with life satisfaction and negatively associated with depression, in line with theoretical expectations. For example, higher acceptance of illness has been linked to lower depressive symptoms (Uchmanowicz et al., 2016) and higher life satisfaction (Kocatepe et al., 2020; Kowalewska et al., 2020; Ministry of Health Sri Lanka, 2022).

To sum up, the obtained results suggest that the Sinhala version of the AIS is both reliable and valid for assessing acceptance of illness (see Table 3 for details).

**TABLE 3:** Means, standard deviations, reliability coefficients, and correlations for study variables (*N* = 141).

Variable	*M*	SD	α	1	2	3
1. Acceptance of Illness	25.96	5.43	0.744	1	–	–
2. Satisfaction with life	23.89	6.21	0.854	0.253**	1	–
3. Depression	9.11	9.03	0.899	−0.336***	−0.488***	1

**P < 0.01, ***p < 0.001.

## Discussion

The primary aim of the current study was to adapt and examine the psychometric properties of the Sinhala version of the AIS. The PCA findings suggested a one-component structure consistent with the original version of the scale. Although the CFA yielded a mixed pattern of fit indices, the overall results provided partial support for the proposed one-factor measurement model. Furthermore, the Sinhala AIS demonstrated satisfactory internal consistency and showed expected associations with life satisfaction and depression, providing preliminary evidence for its construct validity. Overall, the findings support the reliability of the Sinhala version of the AIS and provide preliminary support for its construct validity among Sinhala-speaking individuals with chronic illnesses.

The Sinhala version of the AIS appears to be a promising tool for research and may have potential applications in clinical settings by enabling culturally sensitive measurement of acceptance of illness among Sinhala-speaking populations of people with chronic illnesses. The scale may assist clinicians in identifying individuals with low acceptance of illness levels who could benefit from psycho-social, or counseling interventions designed to enhance coping and resilience Programs focusing on psychoeducation, mindfulness, and acceptance-based therapy could be particularly beneficial in improving patients’ mental health and quality of life (Gregg et al., 2007). Higher levels of illness acceptance are known to be associated with greater adherence to treatment, better emotional regulation, and improved engagement in rehabilitation (Evers et al., 2001). Furthermore, illness acceptance has also been identified as a key psychological factor influencing adaptation to chronic health conditions, with greater acceptance linked to improved quality of life and wellbeing (Iwanowicz-Palus et al., 2020). Given that chronic illness status in the present study was based solely on participants’ self-identification and was not medically verified, these potential clinical applications should be interpreted with caution. Therefore, assessment of illness acceptance may inform patient-centered care and support holistic management of chronic diseases.

Beyond these clinical implications, socioeconomic context, cultural beliefs, and access to healthcare services may be relevant factors influencing illness acceptance and therefore warrant further investigation. In Sri Lankan society, where collectivist values and religious beliefs often play an important role, qualitative evidence suggests that acceptance may be supported through spiritual meaning-making and social support (Bulathwatta et al., 2025). However, these factors were not examined in the present study and should be explored in future research to better understand cultural influences on psychological adaptation.

### Limitations

This study has several limitations that should be acknowledged. Firstly, the sample contains certain biases. The sample size was limited and may not be representative of the broader population, partly due to the use of snowball sampling and the overrepresentation of students. In addition, men were underrepresented in the sample (36.9%), which may affect the generalizability of the results across genders. Moreover, information on participants’ age is imprecise as age was assessed using categorical ranges instead of exact numerical values. Furthermore, given that computer literacy was required for participation, conducting the study online may have excluded less digitally literate individuals. A further limitation is that chronic disease status was determined solely through participants’ self-identification and self-report. No information regarding medical diagnoses, disease type, disease duration, or medical verification was obtained. Therefore, the heterogeneity of chronic conditions represented in the sample could not be assessed.

Another limitation concerns the linguistic reach of the current adaptation of the AIS and its translation procedure. Since the instrument was translated only into Sinhala, its applicability is limited to Sinhala-speaking communities. Consequently, this limits the cultural and linguistic representativeness of the tool and constrains the generalizability of the findings to populations beyond Sinhala speakers, such as Tamil-speaking individuals, the country’s second-largest ethnolinguistic group.

Although the translation process included independent forward translation and back-translation procedures, more extensive cross-cultural adaptation methods (e.g., expert panel review, cognitive interviewing, and formal content validation) were not conducted.

Some limitations concern the data analysis plan. First, both the PCA and CFA were performed on the same sample, which limits the ability to treat the CFA results as an independent validation of the dimensional structure. Although CFI, TLI, AGFI, and SRMR indicated good fit, the RMSEA value suggested only marginal fit. The study provides preliminary results suggesting the one-factor structure rather than definitively confirming it. Replication in larger and independent samples is required to establish the stability and generalizability of the factor structure. Another limitation is that test-retest reliability was not examined as the study had only one measurement point. Therefore, the temporal stability of the Sinhala version of the AIS remains unknown and should be evaluated in future studies using repeated measurements over an appropriate time interval. Therefore, additional studies that address these limitations could further evaluate the cultural appropriateness, content validity, factor structure, and temporal stability of the Sinhala AIS.

### Future directions

Future research should aim to validate the Sinhala version of the AIS among individuals with specific chronic conditions, such as cancer, psychiatric disorders, or diabetes, to enhance its applicability across diverse clinical populations. Moreover, expanding the validation of the scale to include other language groups in Sri Lanka - particularly the Tamil-speaking community, the country’s second-largest ethnic group and one of its official language communities - would further support the scale’s cultural and linguistic inclusivity. Such efforts would contribute to the development of a more comprehensive assessment of illness acceptance in multicultural and multilingual contexts.

## Data Availability

The raw data supporting the conclusions of this article will be made available by the authors, without undue reservation.
