# Supplementary material for: Adapting the acceptance of illness scale for Sinhala-speaking populations: a psychometric study
Source: Front Psychol. 2026 Sep 23;17:1889860. doi: 10.3389/fpsyg.2026.1889860 (PMC13642704; doi:10.3389/fpsyg.2026.1889860)
Supplement: Supplementary file 1 [file Table_1.docx]

**රෝගය පිළිගැනීමේ පරිමාණය**

නම :

දිනය :

උපදෙස්

කරුණාකර පහත සඳහන් සෑම අයිතමයකටම ප්‍රතිචාර දක්වන්න. ඔබ වඩාත් හොඳින් විස්තර කරන අයිතමයට යාබද පරිමාණයෙන් 1 සිට 5 දක්වා අංකයක් තෝරා ගන්න. ඉන්පසු ඔබ තෝරාගත් අංකය රවුම් කරන්න. කිසිදු ප්‍රශ්නයකට නිවැරැදි පිළිතුරක් නැත.

1. මගේ අසනීපයේ සීමාවන්ට හැඩගැසීමට මට අපහසුයි.

දැඩි ලෙස එකඟ වේ	1	2	3	4	5	දැඩි ලෙස එකඟ නොවේ

2. මගේ අසනීපය නිසා මම වැඩිපුරම කරන්න කැමති දේවල් මට මග හැරුණි.

දැඩි ලෙස එකඟ වේ	1	2	3	4	5	දැඩි ලෙස එකඟ නොවේ

3. මගේ අසනීපය නිසා ඇතැම් විටෙක මා කිසි වැඩකට නැති අයෙකු ලෙස දැනේ.

දැඩි ලෙස එකඟ වේ	1	2	3	4	5	දැඩි ලෙස එකඟ නොවේ

4. සෞඛ්‍ය ගැටලු නිසා මට ඕනෑවට වඩා අන් අය මත යැපීමට සිදු වේ.

දැඩි ලෙස එකඟ වේ	1	2	3	4	5	දැඩි ලෙස එකඟ නොවේ

5. මගේ අසනීපය මගේ පවුලට සහ මිතුරන්ට බරකි.

දැඩි ලෙස එකඟ වේ	1	2	3	4	5	දැඩි ලෙස එකඟ නොවේ

6.මාගේ සෞඛ්‍යය මට්ටමක තිබෙනවායයි හැගීමක් මට ඇත්තේ නැත.

දැඩි ලෙස එකඟ වේ	1	2	3	4	5	දැඩි ලෙස එකඟ නොවේ

7. මට හැගෙන්නේ මා විසින් මාගේ සතුට සොයාගැනීමට නොහැකිවේයයි කියාය.

දැඩි ලෙස එකඟ වේ	1	2	3	4	5	දැඩි ලෙස එකඟ නොවේ

8.මට හැගෙන්නේ මාගේ අසනීපය නිසා අනෙක් අය මාගෙන් දුරස් වනවාය කියාය.

දැඩි ලෙස එකඟ වේ	1	2	3	4	5	දැඩි ලෙස එකඟ නොවේ
